# Predictors and consequences of homelessness in whole-population observational studies that used administrative data: a systematic review

**DOI:** 10.1186/s12889-023-16503-z

**Published:** 2023-08-24

**Authors:** Eileen Mitchell, Tanisha Waring, Elayne Ahern, Diarmuid O’Donovan, Dermot O’Reilly, Declan T. Bradley

**Affiliations:** 1 Centre for Public Health, Queen’s University Belfast, Belfast, UK; 2 Public Health Agency, Belfast, UK; 3 University of Limerick, Limerick, Ireland

**Keywords:** Homelessness, Administrative data, Observational studies, Healthcare, Record linkage, Data linkage, PRISMA, Systematic review

## Abstract

**Background:**

Homelessness is a complex societal and public health challenge. Limited information exists about the population-level health and social care-related predictors and consequences of persons with lived experience of homelessness (PEH). Studies that focus on population subgroups or ad hoc questionnaires to gather data are of relatively limited generalisability to whole-population health surveillance and planning. The aim of this study was to find and synthesise information about the risk factors for, and consequences of, experiencing homelessness in whole-population studies that used routine administrative data.

**Method:**

We performed a systematic search using EMBASE, MEDLINE, the Cochrane Library, Cumulative Index to Nursing and Allied Health Literature (CINAHL) and PsycINFO research databases for English-language studies published from inception until February 2023 that reported analyses of administrative data about homelessness and health and social care-related predictors and consequences. We followed the Preferred Reporting Items for Systematic Reviews and Meta-Analyses (PRISMA) guidelines.

**Results:**

Of the 1224 articles reviewed, 30 publications met the inclusion criteria. The included studies examined a wide range of topic areas, and the homelessness definitions used in each varied considerably. Studies were categorised into several topic areas: Mortality, morbidity and COVID-19; health care usage and hospital re-admission; care home admission and shelter stay; and other (e.g. employment, crime victimisation). The studies reported that that the physical and mental health of people who experience homelessness was worse than that of the general population. Homeless individuals were more likely to have higher risk of hospitalisation, more likely to use emergency departments, have higher mortality rates and were at greater risk of needing intensive care or of dying from COVID-19 compared with general population. Additionally, homeless individuals were more likely to be incarcerated or unemployed. The effects were strongest for those who experienced being homeless as a child compared to those who experienced being homeless later on in life.

**Conclusions:**

This is the first systematic review of whole-population observational studies that used administrative data to identify causes and consequences associated with individuals who are experiencing homelessness. While the scientific literature provides evidence on some of the possible risk factors associated with being homeless, research into this research topic has been limited and gaps still remain. There is a need for more standardised best practice approaches to understand better the causes and consequences associated with being homeless.

**Supplementary Information:**

The online version contains supplementary material available at 10.1186/s12889-023-16503-z.

## Introduction

Homelessness is a major social and economic problem that currently affects an estimated 1.6 billion people worldwide [1–3]. Homelessness statistics differ in many countries, for instance, in the UK, homelessness and housing are devolved matters, meaning that decision-making is split between different parliament areas within the UK such as the Scottish Parliament, the Assemblies of Wales, Northern Ireland and London or to Local Authorities. This can therefore make it difficult to have comparable statistics. Homelessness data is often collected through administrative systems which were built using definitions based on each country’s legislation, and so data are not currently comparable [4–6]. Homelessness affects a wide range of people, covering not just people sleeping rough, but also those in temporary accommodation, sleeping temporarily at friends’ houses, living in unfit dwellings and those threatened with homelessness. The prevalence of conditions such as some infectious diseases, substance abuse, psychiatric disorders and mental illnesses in people who experience homelessness has been reported to be higher than in the wider population [7–9]. For instance, in the UK, a recent study found that 41 per cent of people experiencing homelessness reported a long-term physical health problem and 45 per cent had a diagnosed mental health problem, compared with 28 per cent and 25 per cent, respectively, in the general population [10]. Determining accurate national estimates of homelessness prevalence and health and social characteristics among people experiencing homelessness is difficult for many reasons, including the mobility of the population and a lack of standardised collection of homelessness status in national administrative healthcare data systems [11–15]. A number of record linkage and administrative data research studies [16–19] have been conducted in this area. To our knowledge, there has been no previous review that aimed to synthesise these reports to give an overview of current evidence. This review will be a useful guidance tool for policymakers and practitioners when designing policy or interventions to support people who experience homelessness.

We report a comprehensive overview of the published administrative data research on risk factors associated with experiencing homelessness, and the health consequences of homelessness. First, we provide a high-level descriptive summary overview of the findings.

## Methods

### Search terms

We performed a systematic literature review, searching the following databases: EMBASE, MEDLINE, Cochrane Library, Cumulative Index to Nursing and Allied Health Literature (CINAHL) and PsycINFO. A common set of search terms relating to population, setting, study duration period and design was used in each database, as well as subject headings specific to each database (Supplementary Table (S3)). Search terms included housing status (homeless, homelessness, transient living, street people), and terms related to administrative data (public sector data, record linkage, linking data, administrative data). The Boolean operators “AND” and “OR” were used to combine terms. No limits were set with regards to year of publication or country of origin. Unpublished or grey literature was not included. The search was limited to only include studies in English. The systematic review was registered on Prospero (CRD42022273801).

### Study selection and data extraction

Study eligibility was assessed by title and abstract screening, performed by two independent reviewers. All citations were uploaded into Covidence™ (Veritas Health Innovation, 2016), a cloud-based software program used to organize abstracts and systematic and scoping literature reviews. For the first level of screening, two authors (EM and TW) independently conducted the initial searches (identification and screening).

Articles passing the initial screening were retrieved for a detailed full text evaluation. Two authors (EM and TW) assessed the full-text articles for eligibility and extracted information from each study. A third independent reviewer (DB) was consulted in any case of disagreement. A data extraction sheet was developed, pilot-tested on five eligible studies and refined accordingly. The extracted information included: author(s) information and year of publication; country of publication; study design; linked data sources; statistical analysis and software methods used; funding source; Risk ratios (and 95% Confidence Intervals [CIs]) or equivalent ratios (e.g., incidence rate ratios, standardised mortality ratios) and reported main findings. The information was categorised into the following five subgroups: mortality and morbidity; healthcare utilization; hospital re-admission; care home admission and shelter usage; and other (which included employment, crime victimisation and COVID-19 infection).

### Quality assessment

A review of quality assessment tools was consulted to identify a suitable tool for the present study. Several minimum requirements for a quality assessment tool to be considered ‘good’ have been recommended [20]. We selected checklists for cohort and case–control studies from the Scottish Intercollegiate Guidelines Network (SIGN) for quality assessment of eligible studies [20, 21] because they fulfilled most of these minimum requirements (e.g., methods for selecting study participants; methods for measuring exposure and outcome variables; design-specific sources of bias; methods to control confounding; statistical methods; relatively specific and simple; and showing evidence of careful development). The quality of eligible studies was assessed by one author (EM) and cross-checked by a second author (TW). Inconsistency in quality outcome was resolved by consensus.

## Results

### Study selection

**Fig. 1:**
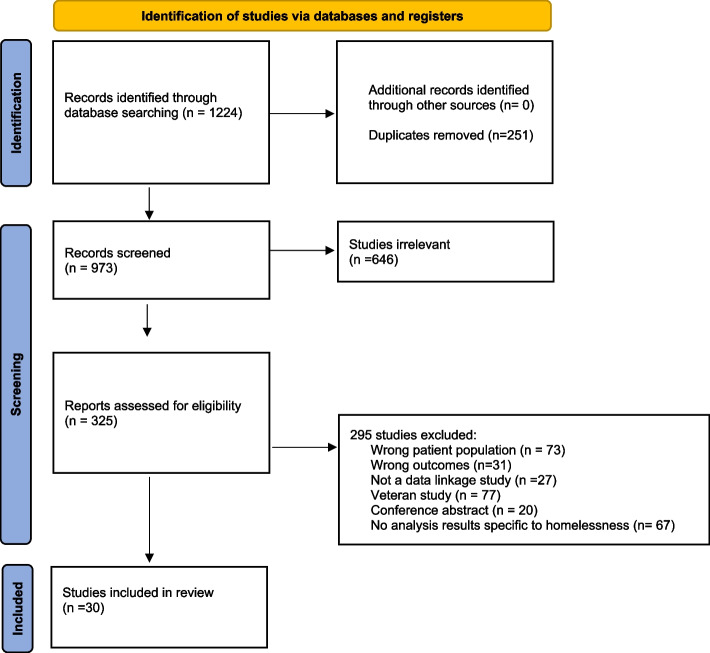
PRISMA flowchart of the study selection process

Searches yielded 1224 articles. After the removal of duplicates, 973 articles remained for screening on title and abstract. 325 articles were screened full-text for relevancy, of which 30 articles were included in the final analysis. The flow of articles retrieved through electronic searches is shown in Fig. 1**.**

### Study characteristics

#### Country origin and publication year

**Fig. 2:**
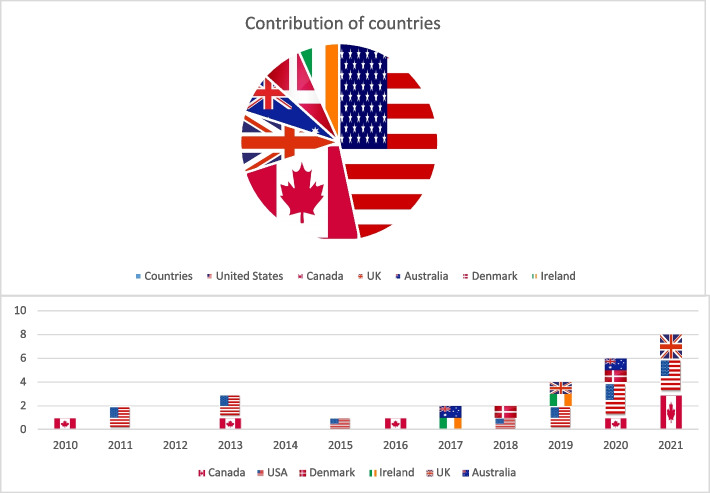
Contributions of countries

Fourteen (46.7%) of studies originated in the USA [22–35], seven (23.3%) in Canada [36–42], three (10.0%) in the UK [43–45], two (6.7%) in Denmark [46, 47], two (6.7%) in Australia [48, 49] and two (6.7%) in Ireland [50, 51]. Seven (23.3%) studies were published before 2015. Thirteen (43.3%) of studies were carried out in hospitals, fourteen (46.7%) were carried out in the community, and three (10.0%) were carried out in emergency shelters, transitional housing facilities, and other social service settings. When the studies reported it, the participants’ mean age ranged from 30 to 61 years of age, with the median age being 43.7 years old. The median percentage of males was 65% and the median number of participants in each study was 2,579. Figure 2 displays the breakdown by country and publication date.

### Outcomes of interest

Six (20.0%) studies [23, 27, 33, 43, 46, 49] had a main outcome of interest of mortality rates in the people experiencing homeless, five (16.7%) studies focused on emergency department (ED) use, three (10.0%) studies focused on hospital readmission, five (16.7%) studies focused on both emergency department (ED) use and hospital readmission, four (13.3%) studies’ outcome of interest was healthcare service usage and two (6.7%) studies reported emergency shelter use. Other outcomes of interest included crime levels, employment rates, underlying cause of death, housing status and COVID-19 infection.

### Statistical analysis and models

Fifteen studies (50.0%) used multivariable logistic regression analyses, four (13.3%) studies used Cox regression models, two (6.7%) studies used a negative binomial model, two (6.7%) studies used crude mortality rates, other methods used included log-likelihood estimation, K-means cluster analysis, Cochran-Armitage test and general linear models.

### Data linkage methods

Twenty-two (73.4%) studies used deterministic linkage methods (using a unique identifier, such as a social security number), four (13.3%) studies used fuzzy or probabilistic linkage (using identifying characteristics) and four (13.3%) studies used both deterministic and probabilistic linkage methods.

### Funding, data sources and software

Twenty (66.7%) of the studies received public grant funding (including government funding), two (6.7%) were university grants and eight (26.7%) studies did not state any type of funding. Seventeen (56.7%) studies linked two data sources, ten studies (33.3%) linked three to five data sources and three (10.0%) studies linked more than five data sources. Thirteen (43.3%) studies used SAS software, five (16.7%) used SPSS, three (10.0%) used Stata, two (6.7%) used R, three (10.0%) used a combination of either R, STATA and SPSS and four (13.3%) did not state which software they used.

### Assessment of study quality

The quality assessment indicated that most included studies were of acceptable quality. No study met all the methodological criteria. The average mean score was 9.3 points (range 7–12, with higher scores denoting higher quality articles). Most studies clearly stated the research objectives, presented sufficient sample sizes for the analyses they undertook, and most articles described the study settings in detail. The lowest scores were obtained in the questions referring to methods to assess the outcome measures, and the appropriateness of the statistical analysis used.

### Synthesis of results

Table 1 summarises the characteristics of included studies. The studies reviewed evaluated a wide range of whole population observational studies that used administrative data. The studies were categorised according to their main outcome areas of interest, these included: (1) Mortality, Morbidity and COVID-19 infection levels. The main outcome we were interested in these studies related to mortality rates and causes of deaths amongst people who experienced homelessness, as well as overall and cause-specific mortality and COVID-19 infection levels and associated deaths. (2) Health care usage, these studies primarily focused on how the people who experienced homelessness were using health services. (3) Hospital re-admission, these studies focused on persons with lived experience of homelessness who had had been discharged from a hospital and been admitted again. Readmission rates have increasingly been used as an outcome measure in health services research and as a quality benchmark for health systems. (4) Care home admission and shelter stay, these studies, these studies focused on homeless individuals who had been admitted to refuge shelters, nursing homes, care homes, temporary accommodation shelters and (5) Other, these studies focused on other non-health related issues such as associations with being homeless and employment levels, and being the victim of criminal offences.

**Table 1:** Provides a summary of the characteristics of the studies included in this review

	**Included studies (*n* = 30) n (%)**	**References**
**Characteristics of participants, where reported in studies**		
Median no. of participants	2579	
Median % male	65	
Median age of participants	43.7	
**Year of study**		
2010 – 2012	3 (10.0)	[27, 33, 36]
2013 – 2014	3 (10.0)	[30, 34, 37]
2015 – 2016	2 (7.0)	[26, 41]
2017 – 2018	4 (14.0)	[23, 47, 48, 50]
2019 – 2020	10 (33.0)	[22, 25, 28, 31, 32, 39, 43, 46, 49, 51]
2021	8 (28.0)	[24, 29, 35, 38, 40, 42, 44, 45]
**Country**		
United States	14 (47.0)	[22–35]
United Kingdom	3 (10.0)	[43–45]
Canada	7 (23.0)	[36–42]
Australia	2 (7.0)	[48, 49]
Denmark	2 (7.0)	[46, 47]
Ireland	2 (7.0)	[50, 51]
**Time horizon**		
1 year or less	7 (23.0)	[24, 26, 28, 30, 40, 41, 50]
2—3 years	5 (17.0)	[22, 32, 43, 44, 48]
4—5 years	8 (27.0)	[34, 36, 37, 39, 42, 45, 46, 51]
5 + years	10 (33.0)	[23, 25, 27, 29, 31, 33, 35, 38, 47, 49]
**Focus area**		
Mortality, morbidity and COVID-19	7 (23.3)	[23, 27, 33, 40, 43, 46, 49]
Healthcare utilisation	12 (40.0)	[22, 24–26, 29, 34, 36, 37, 39, 42, 45, 50]
Care home admission and shelter usage	3 (10.0)	[35, 38, 51]
Hospital re-admission	6 (20.0)	[28, 30–32, 41, 44]
Other (employment rates and police crime victimisation)	2 (6.7)	[47, 48]
**Number of linked data sources used**		
Less than 2	17 (57.0)	[25–30, 32, 34, 35, 38, 39, 41, 45, 48, 49, 51]
3 to 5	10 (33.0)	[23, 31, 33, 36, 37, 43, 44, 46, 47, 50]
Greater than 5	3 (10.0)	[24, 40, 42]
**Data linkage method**		
Deterministic linkage (using an unique identifier, such as a social security number)	22 (73.0)	[25–28, 31, 32, 34–38, 40–45, 47, 49–51]
Fuzzy / Probabilistic linkage (linking by people's names etc.)	4 (13.0)	[29, 30, 33, 48]
Deterministic and Probabilistic	4 (13.0)	[22–24, 39]
**Software**		
SPSS	5 (17.0)	[22, 36, 45, 50, 51]
STATA	3 (10.0)	[23, 26, 28]
R	2 (7.0)	[35, 44]
SAS	13 (43.0)	[24, 25, 29–34, 37, 40–42, 47]
Stata and R	1 (3.0)	[43]
SPSS and Stata	1 (3.0)	[49]
SAS and R	1 (3.0)	[39]
Not reported	4 (13.0)	[27, 38, 46, 48]
**Source of funding**		
Public grants (including government)	20 (67.0)	[23, 25, 33–38, 40, 42–48, 50]
University grants	2 (7.0)	[28, 29]
not stated	8 (27.0)	[22, 26, 27, 30, 32, 38, 49, 51]

### Mortality, morbidity and COVID-19

Seven studies [23, 27, 33, 40, 43, 46, 49] (23%) examining mortality and morbidity in people who experienced homelessness were identified. Three studies were conducted in the United States [23, 27, 33], one in the UK [43], one in Australia [49], one in Denmark [46] and one in Canada [40]. Mortality outcomes studies included mortality rates and causes of deaths, as well as overall and cause-specific mortality. For instance, a study by Aldridge et al. (2021) [43] examined the contribution of different causes of death to overall mortality in PEH recently admitted to hospitals in England with specialist integrated homeless health and care (SIHHC) schemes. The authors found that amongst individuals who experienced homelessness median age of death was 51.6 years (interquartile range [IQR] 42.7–60.2) for SIHHC, compared with a median age of death of 71.5 (IQR 60.67–79.0) in areas of high social deprivation (index of multiple deprivation 5^th^ quintile, IMD5). The top three most frequent underlying causes of death by ICD-10 chapter in the SIHHC group were external causes of death (21.7%), followed by cancer (19.0%) and lastly digestive disease (19.0%). The authors also noted that around one third of deaths in the PEH group were from conditions amenable to healthcare, highlighting an opportunity to intervene in a timely manner. They recommended that more research was need to identify individuals at risk of experiencing homelessness earlier and develop models of care that enable them to engage with interventions proven to either prevent or improve outcomes for early-onset chronic disease. Seastres et al. (2021) [49] examined the long-term effects of ever experiencing homelessness on mortality over a 15-year period in Australia. Findings from the study found that PEH had an increased mortality rate (11.9 vs. 8.1 per 1,000 person-years), significantly higher mortality risk (rate ratio 1.47, 95% confidence interval [CI] 1.26–1.71) and a lower median age at death (66.60 vs. 78.19 years) compared to individuals who had not experienced homelessness. The authors concluded that more research was need to accurately identify individuals experiencing primary, secondary or tertiary homelessness at the emergency department may enable targeted interventions that could potentially reduce their risk of premature mortality. Richard et al. (2021) [40] described and compared testing for SARS-CoV-2, test positivity and hospital admission, receipt of intensive care and mortality rates related to COVID-19 for people with a recent history of homelessness versus individuals who live in a community dwelling (people who live in the community on their own as opposed to those taken care of in nursing homes). The authors found that people with a recent history of homelessness were more likely to be tested for SARS-CoV-2 compared with community-dwelling people (pre-lockdown) adjusted HR 1.61, (1.22–2.11); peak lockdown, adjusted HR 2.95, (2.88–3.03); reopening adjusted HR 1.45, (1.39–1.51). Additionally, PEH individuals were more likely to have a positive test result (peak adjusted HR 3.66, (3.22–4.16)); reopening adjusted HR 1.76, 95% (1.15–2.71)). During the peak period, individuals with a recent history of homelessness were more than 20 times more likely to be admitted to hospital for COVID-19 (adjusted HR 20.35, (16.23–25.53)), on average 10 times more likely to need intensive care as a result of COVID-19 (adjusted HR 10.20, (5.81–17.93)) and more than 5 times more likely to die within 21 days of their first positive test result (adjusted HR 5.73, (3.01–10.91)).

### Healthcare usage

Twelve studies (40%) investigating health care usage were identified [22, 24–26, 29, 34, 36, 37, 39, 42, 45, 50]. Healthcare usage includes ED usage, GP attendance, outpatient visits and costs such as total healthcare costs and pharmacy costs. Of these, six were conducted in the USA [22, 24–26, 29, 34], four in Canada [36, 37, 39, 42], one in Ireland [50] and one in the United Kingdom [45]. The duration of these studies was between 1 and 7 years. Wiens et al. (2021) [42] conducted a longitudinal data linkage study that examined the association between housing status and health care usage in a sample of adults who experienced homelessness in Ontario. The main findings of the study demonstrated that overall, prescription usage was higher for those persons who were inconsistently housed (RR = 1.49; (1.27–1.76)), compared to those who were housed. With regards to overall healthcare usage costs, the highest costs per person-year occurred during person-years spent homeless ($8195; ($7326–$9063)), with lower healthcare costs being reported for those inconsistently housed ($6624; ($6128–$7121)) and housed ($6605; ($6251–$6959)). The authors concluded that providing individuals who experience homelessness with suitable housing may be a suitable option for reducing health care usage costs over time. Paudyal et al. (2021) [45] aimed to identify the demographic characteristics and clinical reasons for all visits made by persons experiencing homelessness (PEH) over a 5-year period at a major ED in the West Midlands. The authors noted that over the course of the study period, around 74% of PEH attendees were male. Alcohol and drug related conditions, as well as injury and pain were the most frequent reasons for PEH individuals requiring ED attention. The authors also noted that a significantly higher proportion of males presented with alcohol and drug problems than females. The observed rate of death of PEH in ED was found to be on average 12 times higher than the rest of the general population. A very high proportion of PEH individuals also left the ED before being treated. A study by Wadhera et al. (2019) [25] reported similar findings. The authors evaluated patterns, causes, and outcomes of acute hospitalisation among PEH compared with a demographics-standardized and risk-standardized non-homeless cohort. They found that hospitalisations for PEH individuals, compared with demographics-standardised non-homeless, were more frequent for substance use and mental illness (52% vs. 18%, *P* < 0.001). People with lived experience of homelessness compared to the rest of the population on average had lower in-hospital mortality rates (0.9% vs. 1.2%, *P* < 0.001), longer mean length of stay (6.5 vs. 5.9 days, *P* < 0.001), and lower mean costs per day ($1,535 vs. $1,834, *P* < 0.001). The findings from these studies indicate that there is substantial literature on the association between homelessness and substance and/or alcohol dependence; these issues were cited as both cause and consequences of homelessness [30, 37, 52–54]. These studies further demonstrate that these issues contribute to PEH- most frequent reasons for usage of emergency health care.

### Hospital re-admission

Six studies (20%) assessed hospital re-admission [28, 30–32, 41, 44]. We define a hospital readmission as an episode in which a patient who had previously been discharged from a hospital is admitted again within a specified time interval. Of these studies, four were conducted in the United States [28, 30–32], one in Canada [41] and one in the UK [44]. The duration of these studies was between 1 and 6 years. The two main outcome measures reported in all studies included ED usage and hospital re-admission rates. However, evidence about whether they experience higher re-admission rates, as well as if readmissions vary by region or cause of hospitalisation, is limited. Lewer et al. (2021) [44] compared the risk of hospital re-admission among PEH with housed inpatients living in socioeconomically deprived areas. After adjusting for health measured at the index admission, PEH had 2.49 (2.29 to 2.70) times the rate of emergency re-admission, 0.60 (0.53 to 0.68) times the rate of planned re-admission and 2.57 (2.41 to 2.73) times the rate of Accident and Emergency (A&E) visits compared with housed patients. The 12-month risk of emergency re-admission was higher for persons with lived experience of homelessness at 61%, (59% to 64%) than housed patients at 33% (30% to 36%); and the risk of planned re-admission was lower for PEH group at 17%, (14% to 19%) than for housed patients recorded at 30% (28% to 32%). The risk of emergency re-admission was high across all causes for persons with lived experience of homelessness. In contrast, the reason for admissions among housed patients varied with admissions as a result of cancer, for example, being higher than admissions due to accidents. Khatana et al. (2020) [31] reported that homelessness at the time of discharge is associated with significantly higher rates of 30- and 90-day re-admission rates across multiple US states. The adjusted analysis showed that the 30-day and 90-day re-admission rates were higher for index hospitalisations in the homeless group compared with those in the housed group (17.3% (SE = 0.04) vs. 14.0% (SE = 0.02), *p* < 0.001; and 23.8% (SE = 0.05) vs. 19.9% (SE = 0.02), *p* < 0.001, respectively). A similar trend was observed for 90-day re-admissions. Index hospitalisations are the first in a series of hospitalisations that a patient is admitted for a specific condition or diagnosis. The authors reported that the four most common causes for index hospitalisations in the persons with lived experience of homelessness group were mental illness, complications of pregnancy, childbirth and the puerperium, circulatory diseases, and diseases of the digestive system. LaWall et al. (2019) [32] conducted a study that examined if social isolation and homelessness, could be used to predict 30-day potentially preventable re-admission (PPR). The authors found that social isolation was indeed a contributor to all-cause mortality and overall higher health care usage amongst PEH. The authors also noted that a number of additional factors were also found to be significantly associated with 30-day PPR. For instance, those who had received a referral from a physician were less likely to have a 30 day PPR compared to those who were not PEH individuals who were admitted through the emergency department (OR = 0.73; (0.58–0.92). Additionally, PEH who were admitted to a skilled nursing facility were less likely than those who were discharged to home or self-care to have a 30-day PPR (OR = 0.65; (0.51–0.82)). The study also reported that the percentage of people with lived experience of homelessness with comorbidities was significantly higher than for those who were not homeless (*p* < 0.001). A greater percentage of patients who were homeless had four or more comorbidities (76%), compared to those who were not homeless (62%). None of the PEH individuals in the study had zero comorbidities. Miyawaki et al. (2020) [28] investigated whether PEH experience higher rates of readmissions and ED visits after hospital discharge than non-homeless individuals. Persons with lived experience of homelessness had a high-risk of re-admissions (adjusted rate, 27.3% vs. 17.5%; adjusted odds ratio [aOR], 1.93; (1.69–2.21); *p* < 0.001) and ED visits after hospital discharge (37.1% vs. 23.6%; aOR, 1.98; (1.74–2.25); *p* < 0.001) compared with non-homeless persons with lived experience of homelessness. Persons with lived experience of homelessness treated at homeless-serving hospitals had lower rates of readmissions (23.9%vs. 33.4%; *p* < 0.001) and ED visits (31.4% vs. 45.4%; *p* < 0.001) after hospital discharge compared to PEH. The most consistent risk factors for PEH being re-admitted identified by all studies were substance abuse and mental health problems. Substance abuse problems appeared to be the risk factor with the greatest magnitude of effect.

### Care home admission and shelter stay

Three studies [35, 38, 51] (10%) assessed nursing home admission and shelter stay.

Sheltered housing is accommodation specifically designed for older or disabled people to allow them to live independently. It usually consists of self-contained flats with communal facilities. In contrast, nursing homes, which are sometimes referred to as residential nursing homes or care homes with nursing, there is always at least one qualified and registered nurse on site, meaning residents have access to 24-h medical care and skilled nursing support.

Of these, one was conducted in Ireland [51], one in Canada [38] and one in the United States [35]. The duration of these studies was between 4 and 6 years. The three main outcome measures included emergency shelter usage, housing status and predictors of care home admission. Byrne et al. (2021) [35] investigated the extent and timing of nursing care home admissions among older adults who had their first visit at an emergency shelter. Around 12% of the study cohort had a nursing home admission within 4 years of their initial shelter entry. Additionally, the highest risk of nursing home admission occurred during the first few months following shelter entry. A number of factors were associated with having a higher risk of care home admission following shelter entry, these included having an alcohol use disorder, being older, and having had a prior history of care home admission. Waldron et al. (2019) [51] examined the patterns of emergency accommodation use by people experiencing homelessness in Dublin City. The authors found that younger individuals are more likely to require emergency accommodation for short periods of time and transition in and out of homelessness, whilst middle aged adults are more likely to be long term users of emergency accommodation. Chen et al. (2021) [38] compared homelessness pathways and housing outcomes between first-time and recurrent shelter users. The authors reported that recurrent shelter users are more likely to be single, male and aged between 25–54 years old.

### Other (employment rates and crime victimisation)

Several potentially important risk factors for homelessness were identified and measured in two studies. One study focused on people with lived experience of homelessness who have been a victim of criminal offences[47], and another investigated links between homelessness and adult employment [48]. Of these, one was conducted in Denmark [47] and one in Australia [48]. The duration of these studies was between 3 and 15 years. A study by Nilsson et al. (2018) [47] examined the risk of police-recorded crime victimisation in individuals with experiences of homelessness and compared this with the general population in Denmark. The authors reported that compared to the general population, homeless individuals were more likely to be at increased risk of any crime victimisation (incidence rate ratios (IRRs) 2.7 (2.4–3.0)) in females and 2.3 (2.1–2.5) in males), and in particular, violent crime victimisation 7.2 (6.3–8.2) in females and 3.6 (3.2–4.0) in males). The authors noted that individuals who had experienced homelessness and who also had a psychiatric diagnosis had the highest risk of violent victimisation IRR 10. (8.6–11.9) in females and 4.3 (3.8–4.9) in males), compared to those individuals who had no psychiatric diagnosis or experience of homelessness (the reference group). Five years after an individual’s first made contact with a homeless shelter, the cumulative probabilities of any crime victimisation were highest in females at 23% (21–26) and around 16% (15–18) in males, these figures were substantially higher compared to the general population. Cobb-Clark et al. (2017) [48] examined the long-run employment consequences of experiencing homelessness in childhood rather than later in life. The authors reported that men who were first homeless at or before the age of 15 are more than twice as likely (34% vs. 16%) to be incarcerated between the ages of 17 and 20. Whilst individuals who were first homeless in childhood (i.e., at age 15 or below) were significantly less likely to be working in adulthood (aged 18 +).

Additionally, men who experience homelessness as children were around 13% less likely to be employed, this figure was even higher for woman at around 16%. A complete summary of the included study outcomes is included as supplementary material (Supplementary Table (S2)).

## Discussion

In this systematic review, we aimed to summarize and synthesise information about the risk factors for, and consequences of, experiencing homelessness in whole-population studies that used routine administrative data. One of the main striking outcomes when examining the studies included in this review is the paucity of data in relation to health among individuals who are experiencing homelessness. Of the > 1200 articles associated with administrative data, health and homelessness, < 3% met the inclusion criteria for this systematic review.

The review highlighted that overall the physical and mental health of people who are homeless is generally worse than that of the general population. Homeless individuals are more likely to have higher rates of emergency re-admission, hospital usage, and more frequent ED visits compared to housed patients. These findings are consistent with other studies that have examined healthcare implications for person’s with lived experience of homelessness [55, 56]. PEH individuals had a higher mortality risk and younger median age at death compared to non-homeless individuals. In the peak period of COVID-19, people with a recent history of homelessness were 10 times more likely to require intensive care for COVID-19, and over 5 times more likely to die within 21 days of their first positive test result compared to the general population. PEH individuals were also at a higher risk of being incarcerated and had an increased risk of any crime victimisation. Men who experience homelessness as children are less likely to be employed as adults, the employment gap is even larger for women who experience homelessness as children. Evidence suggests that overall individuals who experience homelessness during childhood when foundational cognitive and non-cognitive skills are being formed are more likely to have more severe and long-term consequences compared to those who experience homelessness later on in life. Crime victimisation can result in limited employment prospects, stigma, and limited social housing support which can ultimately increase risk for homelessness. Adverse childhood experiences and other preliminary factors may also set a pathway toward poverty and homelessness.

Overall, the findings suggest that a better understanding of predictors and consequences of homelessness is required, and this understanding could be applied to the development of policy [57] or individual interventions [58] to reduce homelessness and its adverse effects. It is difficult to infer causality and the direction of causality from observational studies. There may not be a clear distinction between correlates, predictors and consequences of homelessness in this complex system. Data about people experiencing homelessness may be prone to less completeness than other population groups because of the challenges associated with identifying individuals with frequently changing residential addresses over time and between datasets [59–61]. There were challenges comparing between studies because of the different definitions of exposures, outcomes, analytical methods and reporting completeness. Most studies provided insufficient information about the classification of deaths; for example, not identifying which ICD codes corresponded to each cause category, or not indicating whether a category was defined in terms of underlying cause only, or both underlying and contributing causes. To our knowledge, this is the first systematic review of the topic, and it has several strengths. First, the main strength of this review is the systematic approach adopted for reviewing the available body of recently published literature on the topic, with an in-depth screening of the records retrieved from several comprehensive databases like Embase. Our review highlights a number of gaps in the research literature in relation to identifying the predictors and consequences of being homelessness using administrative data that should be examined further in future research.

### Limitations

Our review had several limitations. Firstly, limiting the results to papers published in the English language may have excluded relevant studies published in other languages. Secondly, our findings showed high heterogeneity between studies, therefore it was not possible to conduct a meta-analysis. The way in which homelessness was defined varied considerably between studies, the study duration also varied greatly. There was also limited information regarding the transition into and out of homelessness provided making it difficult to draw comparisons between studies.

Analyses of the cost-effectiveness and clinical effectiveness of using data administrative studies [62] to understand the causes and consequences associated with being homeless were beyond the scope of this review, however, would be useful to include in future studies. Finally, although only administrative data studies were included in this review, other study designs may also be useful to examine.

### Implications for clinical care and homeless policy

The information provided in this review indicates that there is a need for greater treatment, support and improvements in health-related outcomes for individuals who are homeless compared to the general population. Policy makers and clinicians should focus ensuring that homeless people are able to receive health care through coordinated treatment and support programs that are specifically adapted to the needs of PEH. Healthcare providers and housing service providers need to examine in greater detail the associated predictors and consequences of being homelessness.

### Implications for future research

The studies reviewed here indicate that there is a need for timely, detailed and robust evidence on the causes and consequences of persons experiencing homelessness, which can be provided by studies that use population-level administrative data. Future research studies should be broadened to reflect the diversity of the homeless population. Researchers should also consider the inclusion and definition of usual care control comparison groups in future studies. The most common comparator group mentioned in the included studies simply stated that it in comparison to the general population. Considering that general population levels may vary between countries in terms of age and income levels, having this additional information provided or a more detailed description would be beneficial for future studies. There is also a need for future studies to separate the relationship between health and the different stages of homelessness, for instance we found no studies that examined the causes of consequences associated with individuals who threatened with becoming homeless. A recent report by the Scottish Government [17] examined the health inequalities of households threatened with homelessness. The report noted that individuals who were at risk of being homeless were more likely to have increased interactions with healthcare services prior to being homeless and that a peak in interactions was seen around the time of the first formal homelessness assessment. This suggests there may be opportunities to detect impending homelessness, and warrants further exploration in other contexts.

The findings from this review also suggest that there is a need in this field for a core outcome set (COS), which is a list of outcomes that researchers should measure and report if they are undertaking a research study in a particular topic. There is also a need for more nuanced, agreed definitions of the term ‘homelessness’, should a universal definition not be possible, as there appeared to be heterogeneity between studies when using this term.

### Supplementary Information

**Additional file 1: Table S1.**

**Additional file 2: Table S2.**

**Additional file 3: Table S3.**

## Data Availability

Available upon request by contacting lead author EM.
