# Supplementary material for: Predictors and consequences of homelessness in whole-population observational studies that used administrative data: a systematic review
Source: BMC Public Health. 2023 Aug 24;23:1610. doi: 10.1186/s12889-023-16503-z (PMC10463451; doi:10.1186/s12889-023-16503-z)
Supplement: Supplementary file 1 — Additional file 1: Table S1. [file 12889_2023_16503_MOESM1_ESM.docx]

# Table S1

# Detailed breakdown of studies.

| **First author, years, (country)** | **Aim of study** | **Design, duration** | **Homeless population group; Sample size** | **% Male** | **Age (mean)** | | **Data linkage method, statistical model** | **Main outcome** | **Results reported by authors** | **Conclusion reported by authors** | **Quality Rating Overall supporting text** |
| --- | --- | --- | --- | --- | --- | --- | --- | --- | --- | --- | --- |
| **Mortality and Morbidity** | | | | | | | | | | | |
| Aldridge 2019, UK | To examine the contribution of different causes of death to overall mortality in homeless people recently admitted to hospitals in England with specialist integrated homeless health and care (SIHHC) schemes. | Cross sectional study, 3 years | Homeless adults aged 18 or older, 3882 | 56.4 | NA | Deterministic linkage (using an unique identifier, such as a social security number) , proportional mortality ratio | | Underlying cause of death | 600 deaths. The median age of death was 51.6 years (interquartile range 42.7-60.2) for SIHHC and 71.5 for the IMD5 (60.67-79.0). The top three underlying causes of death by ICD-10 chapter in the SIHHC group were external causes of death (21.7%; 130/600), cancer (19.0%; 114/600) and digestive disease (19.0%; 114/600). The percentage of deaths due to an amenable cause after age and sex weighting was 30.2% in the homeless SIHHC group (181/600) compared to 23.0% in the IMD5 group (578/2,512). | Nearly one in three homeless deaths were due to causes amenable to timely and effective health care. The high burden of amenable deaths highlights the extreme health harms of homelessness and the need for greater emphasis on prevention of homelessness and early healthcare interventions. | Good |
| Seastres 2020 , Australia | To examine the effect of homelessness on mortality. | Cohort longitudinal study , 15 years | Individuals included in the present study were adults aged 18 years or older who presented at least once for care at the ED between 1 January 2003 and 31 December 2004, 1575 | 74.8 | 41.02 | Deterministic linkage (using an unique identifier, such as a social security number) , Cox regression model | | Mortality IR (per 1000 p-y) | Over 15 years, homeless individuals had a higher mortality rate (11.89 vs. 8.10 per 1,000 person-years), significantly increased mortality risk (rate ratio 1.47, 95% confidence interval [CI] 1.26-1.71) and younger median age at death (66.60 vs. 78.19 years) compared to non-homeless individuals. Using adjusted Cox proportional hazards models, primary (hazard ratio [HR] 2.05, 95%CI 1.67-2.50), secondary (HR 1.60, 95%CI 1.23-2.10) and tertiary (HR 1.72, 95%CI 1.16-2.56) homelessness were independent risk factors for premature mortality | At least one recorded episode of primary, secondary, or tertiary homelessness was associated with premature mortality and younger age at death over a 15-year period. | Good |
| Roncarati 2018 , United States | To assess the mortality rates and causes of death for a cohort of unsheltered homeless adults from Boston, Massachusetts | Cohort longitudinal study , 10 years | adults living outside who were primary care patients of the Boston Health Care for the Homeless Programs (BHCHPs) Street Team, 445 | 72.35 |  | Deterministic and Probabilistic , Crude Mortality Rate per 100.000 Person-years | | Mortality Ratios | The all-cause mortality rate for the unsheltered cohort was almost 10 times higher than that of the Massachusetts population and nearly 3 times higher than that of the adult homeless cohort. Non-Hispanic black individuals had more than half the rate of death compared with non-Hispanic white individuals, with a rate ratio of 0.4. The most common causes of death were non-communicable diseases (e.g., cancer and heart disease),alcohol use disorder, and chronic liver disease | Mortality rates for unsheltered homeless adults in this study were higher than those for the Massachusetts adult population and a sheltered adult homeless cohort with equivalent services | Good |
| Metraux 2011 , United States | To examine mortality among New York City (NYC) homeless shelter users, assessing the relationships between mortality hazard and time in shelter, patterns of homelessness, and subsequent housing exits for both adults in families and single adults. | Cohort longitudinal study , 12 years | Homeless adults aged 18 or older, 160525 | | NA | Deterministic linkage (using an unique identifier, such as a social security number) , Cox regression model | | Mortality rate | Life expectancy was 64.2 and 68.6 years for single adult males and single adult females, respectively, and among adults in families, life expectancy was 67.2 and 70.1 years for males and females, respectively. For both groups, exits to stable housing (subsidized or non-subsidized) were associated with reduced mortality hazard. And while mortality hazard was substantially reduced for the time adults were in shelters, extended shelter use patterns were associated with increased mortality hazard. Differences between single homelessness and family homelessness extend to disparities in mortality rates. | for both subgroups of the homeless population, prompt resolution of homelessness and availability of housing interventions may contribute to reduced mortality | Fair |
| Benjaminsen 2020 , Denmark | To analyse excess morbidity amongst homeless shelter users compared to the general Danish population. | Cohort longitudinal study , 5 years | Homeless adults aged 18+ using shelters, 14730 | 72.8 | NA | Deterministic linkage (using an unique identifier, such as a social security number) , Logistic regression model | | risk of somatic diseases, excess morbidity | The excess morbidity associated with shelter use is substantially lower than in studies that did not include an extensive control. Approximately 80% of excess morbidity amongst shelter users is attributed to other risk factors. A large part of the excess morbidity is explained by substance abuse problems and lack of employment, whilst mental illness, low income, low education, civil status and ethnic minority background explain only a limited part. However, when conducting an extensive control for confounding, a significantly higher morbidity was identified amongst shelter users for infectious diseases, lung, skin, blood and digestive diseases, injuries, and poisoning. | Ill health amongst homeless shelter users is widely explained by substance abuse problems and other risk factors. Nonetheless, for many diseases homelessness poses an additional risk to the health. | Good |
| Baggett 2011 , United States | To assess overall and cause-specific mortality rates in a large cohort of adults who used services provided by BHCHP from 2003 to 2008. | Cohort longitudinal study , 15 years | all adults at least 18 years old who had an in-person encounter at BHCHP between January 1, 2003, and December 31, 2008., 1302 | 66.4 | 41 | Fuzzy / Probabilistic linkage (linking by people's names etc.) , Crude Mortality Rate per 100.000 Person-years | | mortality rate | A total of 1302 deaths occurred during 90,450 person-years of observation. Drug overdose (n=219), cancer (n=206), and heart disease (n=203) were the major causes of death. Drug overdose accounted for one-third of deaths among adults younger than 45 years. Opioids were implicated in 81% of overdose deaths. Mortality rates were higher among whites than non-whites. | The all-cause mortality rate among homeless adults in Boston remains high and unchanged since 1988 to 1993 despite a major interim expansion in clinical services. Drug overdose has replaced HIV as the emerging epidemic | Fair |
| Richard 2021 , Canada | To describe and compare testing for SARS-CoV-2, test positivity and hospital admission, receipt of intensive care and mortality rates related to COVID-19 for people with a recent history of homelessness versus community-dwelling people as of July 31, 2020. | Cohort study , < 1 year | Individuals experiencing homelessness during a health care encounter between Oct. 1, 2018, and July 31, 2020. , 29407 | 68 | 38 | Deterministic linkage (using an unique identifier, such as a social security number) , Cox-proportional hazards model | | the receipt of a SARS-CoV-2 test | People with a recent history of homelessness were more likely to be tested for SARS-CoV-2 in all 3 periods compared with community-dwelling people. They were also more likely to have a positive test result. In the peak period, people with a recent history of homelessness were over 20 times more likely to be admitted to hospital for COVID-19, over 10 times more likely to require intensive care for COVID-19 and over 5 times more likely to die within 21 days of their first positive test result. | In Ontario, people with a recent history of homelessness were significantly more likely to be tested for SARS-CoV-2, to have a positive test result, to be admitted to hospital for COVID-19, to receive intensive care for COVID-19 and to die of COVID-19 compared with community-dwelling people. People with a recent history of homelessness should continue to be considered particularly vulnerable to SARS-CoV-2 infection and its complications. | Good |
| **Healthcare usage** | | | | | | | | | | | |
| Wadhera 2019 , United States | To evaluate patterns, causes, and outcomes of acute hospitalization among homeless persons compared with a demographics-standardized and risk-standardized non-homeless cohort. | Cohort study , 6 years | Homeless adults aged 18 years or older, 202439 | 76.1 | 46.1 | Deterministic linkage (using an unique identifier, such as a social security number) , Logistic regression model | | in-hospital mortality rates | From 2007 to 2013, hospitalisations for the homeless increased in Massachusetts (294 to 420 hospitalisations per 1000 homeless residents), Florida (161 to 240/1000), and California (133 to 164/1000). Homeless patients were on average 46 years of age, often male (76.1%), white (62%), and either uninsured (41.9%) or insured by Medicaid (31.7%). Hospitalisations for homeless persons, compared with demographics-standardized non-homeless, were more frequently for mental illness and substance use disorder (52% vs. 18%, P < 0.001). Homeless compared with risk-standardized non-homeless individuals had lower in-hospital mortality rates (0.9% vs. 1.2%, P < 0.001), longer mean length of stay (6.5 vs. 5.9 d, P < 0.001), and lower mean costs per day ($1 535 vs. $1 834, P < 0.001). | Hospitalisations among homeless persons are rising. Despite greater policy and public health focus over the last few decades, mental illness and substance use remain primary drivers of acute hospitalization among homeless adults. Policy efforts should address barriers to the use of ambulatory care services, and behavioural health services in particular, to help reduce acute care use and improve the long-term health of homeless individuals | Good |
| Wiens 2021 , Canada | To investigate the relationship between housing and healthcare usage among adults with a history of homelessness in Ontario | longitudinal study , 4 years | Individuals meeting the inclusion criteria, 642 | 31 | 43.7 | Deterministic linkage (using an unique identifier, such as a social security number) , Negative Binomial model | | Ambulatory care visits, prescription medication claims, laboratory billings, medical-surgical hospitalisations, psychiatric hospitalisations, and ED visits. | Over the study period, the proportion of individuals who were housed increased from 37% to 69%. The unadjusted rates of ambulatory care visits, prescription medications, and laboratory tests were highest during person-years spent housed or inconsistently housed and the rate of emergency department visits was lowest during person-years spent housed. Following adjustment, the rate of prescription claims remained higher during person-years spent housed or inconsistently housed compared with the homeless. Rate ratios for other health care encounters were not significant (P>0.05). An interaction between time and housing status was observed for total healthcare costs; as the percentage of days housed increased, the average costs increased in year 1 and decreased in years 2-4. | These findings highlight the effects of housing on health care encounters and costs over a 4-year study period. The rate of prescription medications was higher during person-years spent housed or inconsistently housed compared with the homeless. The cost analysis suggests that housing may reduce health care costs overtime; however, future work is needed to confirm the reason for the reduction in total costs observed in later years. | Good |
| Wang 2015 , United States | to determine whether inappropriate ED use for nonemergency care may be reduced by providing charity insurance and assigning homeless patients to a primary care physician (PCP) in an outpatient clinic setting. | Cohort study , 1 year | homeless patients who presented at the ED in our publicly funded, level I trauma center and teaching county hospital, 867 | 57.09 | 44.54 | Deterministic linkage (using an unique identifier, such as a social security number) , Analysis of variance with Bonferroni correction | | ED use | Following New York University ED Algorithm standards, 76% of all ED visits were deemed inappropriate with approximately 77% of homeless patients receiving charity care and 74% of patients with no insurance seeking non crisis health care in the ED (P= .112). About 50% of inappropriate ED visits and 43.84% of appropriate ED visits occurred in patients with a PCP assignment (P=0.019) | Both charity care homeless patients and those without insurance coverage tend to use the ED for non-crisis care resulting in high rates of inappropriate ED use. Simply providing charity care and/or PCP assignment does not seem to sufficiently reduce inappropriate ED use in homeless patients | Good |
| Trick 2021 , United States | To evaluate the variability of health system use; healthcare fragmentation across systems; and medical co-morbidities across the spectrum of homeless typologies. | Cross sectional study , 1 year | Homeless individuals with a healthcare encounter, 11447 | 68.2 |  | Deterministic and Probabilistic , Cochran-Armitage test | | Health service use | Among 11,447 homeless recipients of healthcare, nearly 1 in 5 were identified by ICD10 code alone without recorded homeless services (n= 2177; 19%). Almost half received homeless services that did not include stable housing (n= 5444; 48%), followed by stable housing (n= 3017; 26%), then receipt of both stable and unstable services (n= 809; 7%) | Differences in behavioural-health conditions and health-system use across homeless typologies highlight the particularly high burden among homeless who are disconnected from homeless services. Fragmented and high use of emergency departments for care should motivate health systems and payers to promote housing solutions, especially those that incorporate substance use and mental health treatment. | Good |
| Petrovich 2020 , United States | To identify differences in demographic characteristics, health service use, and predictors of health service use among people experiencing unsheltered and sheltered homelessness. | Cohort study , 2 years | Homeless adults aged over 18, 740 | 64.7 | 48.83 | Deterministic and Probabilistic , Logistic regression model | | Predictors of Service Utilisation | Compared to sheltered participants, unsheltered participants had higher proportions of males and Caucasians, were younger, were more likely to use any type of health service and ED services, and used significantly more of any health service and ED and outpatient services. Results also confirm that health services utilisation is a complex phenomenon predicted by a variety of predisposing, enabling, and need-related factors, including mental health problems. | these findings demonstrate important differences between people living unsheltered and those residing in shelters and they inform local health policy and program initiatives tailored towards these homeless populations | Good |
| Paudyal 2021 , UK | To identify the demographic characteristics and clinical reasons for all visits made by Persons experiencing homelessness (PEH) over a 5-year period at a major ED in the West Midlands. | Cohort study , 5 years | Homeless adults aged 18+, 3271 | 74.1 | 39 | Deterministic linkage (using an unique identifier, such as a social security number), multivariate analyses | | Clinical characteristics, attendance outcomes and deaths | During the study period, 3271 of 596,198 presentations were made by PEH; 74% PEH attendees were male. Drug- and alcohol-related conditions, as well as pain and injury constituted the most frequent reasons for presentation, contributing to over half of all presentations. A significantly higher proportion of males (n=481, 20.3%) presented with drug and alcohol problems than females (n=93, 11.2%). | Drug, alcohol and pain including the need of opioid analgesics constituted the majority of presentations made by PEH in ED. The observed rate of death of PEH in ED is 12 times higher than the general population. A very high proportion of PEH also leave the ED before being treated. pain was the primary reason for presentation for twice as many female patients. Nearly one in five left the ED before being assessed and a total of 39 patients (1.2%) died in the ED and 785 (24.0%) required in-patient admissions to the same hospital. | Good |
| Madigan 2021 , United States | To describe individuals coded as homeless in state-level data comprising of outpatient and inpatient cases over a multi-year period to provide public health surveillance data on the health care utilisation and needs of this population. | Cross sectional study , 7 years | All individuals meeting definition of homelessness, 154173 | | 44 | Fuzzy / Probabilistic linkage (linking by people's names etc.) , Logistic regression model | | Predictors of discharge to a health care facility versus routine discharge to home or self-care | There were 154,173 patient visits predominantly involving males, those aged 25 - 64 years, and non-Hispanic Whites and African Americans. The majority had comorbidities of depression, psychosis, and/or substance abuse (70.2%) and a routine discharge to home or self-care (81.9%). Discharge to home or self-care relative to another health care institution was associated with having charity coverage and being Black/African American. | Those experiencing homelessness experience a high burden of health concerns. Hospital billing records can be used to prioritize the distribution of limited public health resources for healthcare programs and interventions among those experiencing homelessness | Fair |
| Gadermann 2020 , Canada | To examine the association of residential instability with hospitalisations among homeless and vulnerably housed individuals over a 4-year time period. | Cohort study , 4 years | individuals who were homeless or vulnerably housed in three Canadian cities from 2009 to 2013, 378 | 65.1 | 42.3 | Deterministic and Probabilistic , Logistic regression model | | whether or not participants had experienced any hospitalization during the 12 months prior to the baseline interview or the interval period between follow-up interviews | A higher number of residential moves were associated with hospitalization over the study period. Transgender, female gender, perceived social support, better self-reported mental health, and having chronic health conditions also predicted having been hospitalized over the study period, whereas high school/higher education was negatively associated with hospitalisations | Residential instability is associated with increased risk of hospitalization, illustrating the importance of addressing housing as a social determinant of health. | Good |
| Cheallaigh 2017 , Ireland | To compare the use of unscheduled emergency department (ED) and inpatient care between housed and homeless hospital patients in a high-income European setting in Dublin, Ireland. | Cross sectional study , 1 year | Homeless population of the catchment area of St. James's Hospital, Dublin., 2966 | 51.5 | NA | Deterministic linkage (using an unique identifier, such as a social security number) , multivariate analyses | | ED attendances and unscheduled inpatient stay | In comparison with housed individuals in the hospital catchment area, homeless individuals had higher rates of ED attendance. The rate of leaving ED before assessment was higher in homeless individuals. Homeless patients were more likely to terminate an inpatient admission against medical advice | Homeless patients represent a significant proportion of ED attendees and medical inpatients. In contrast to housed patients, the bulk of usage of unscheduled care by homeless people occurs in individuals aged 25 - 65 years | Good |
| Chambers 2013 , Canada | To identify predictors of emergency department (ED) use among a population-based prospective cohort of homeless adults in Toronto, Ontario | Cross sectional study , 4 years | Single adult men, single adult women, and family adults experiencing homelessness, 1165 | | 36.1 | Deterministic linkage (using an unique identifier, such as a social security number) , Logistic regression model | | Frequent emergency department (ED) use among homeless persons | Among 1165 homeless adults, 892 (77%) had at least 1 ED visit during the study. The average rate of ED visits was 2.0 visits per person-year, whereas frequent users averaged 12.1 visits per person-year. Frequent users accounted for 10% of the sample but contributed more than 60% of visits. Predictors of frequent use in adjusted analyses included birth in Canada, higher monthly income, lower health status, perceived unmet mental health needs, and perceived external health locus of control from powerful others; being accompanied by a partner or dependent children had a protective effect on frequent use | Among homeless adults with universal health insurance, a small subgroup accounted for the majority of visits to emergency services. Frequent use was driven by multiple predisposing, enabling, and need factors | Good |
| Brown 2013 , United States | To compare the characteristics of emergency department (ED) visits of older versus younger homeless adults. | Cohort study , 4 years | homeless patients aged 18 years and older, 560 510 ED visits | 74 |  | Deterministic linkage (using an unique identifier, such as a social security number) , multivariate analyses | | characteristics of ED visits | The ED visits of homeless adults aged 50 years and older accounted for 36% of annual visits by homeless patients, Older homeless adults had fewer discharge diagnoses related to psychiatric conditions and drug abuse but more diagnoses related to alcohol abuse and were more likely to arrive by ambulance and e admitted to the hospital | Older homeless adult’s patterns of ED care differ from those of younger homeless adults. Health care systems need to account for these differences to meet the needs of the aging homeless population | Good |
| Bonin 2010 , Canada | To describe the service utilisation by clients of homeless resources in Quebec and Montreal (Canada) over a 5-year period. | Cohort longitudinal study , 5 years | Participants were recruited by research assistants who went to the resource centres and surveyed clientele to identify those who met the research criteria, 426 | 87.4 | NA | Deterministic linkage (using an unique identifier, such as a social security number) , multivariate analyses | | Utilisation of services | in general, mental health services are less used than physical health services; women, older people, and persons with mental health problems used these services more frequently, participants involved in this study tend to continue using services over years in a system where health services are free. | the service utilisation portrait of persons with concomitant problems remains a complex issue and certainly requires further investigation | Good |
| **Hospital re-admission** | | | | | | | | | | | |
| Saab 2016 , Canada | To compare the hospital readmission rate among individuals experiencing homelessness with that of a low-income matched control group, and to identify risk factors associated with readmission within the group experiencing homelessness. | Cohort study , 1 year | residing in a shelter, public place, abandoned building, or vehicle, or staying temporarily with another person (couch-surfing) within the previous seven days, and not having a place, 203 | 60.1 | 40.9 | Deterministic linkage (using an unique identifier, such as a social security number) , multivariate analyses | | The occurrence of an unplanned medical or surgical readmission within 30 days of discharge from hospital. | Between 6 December 2004 and 31March 2009, homeless participants (N=203) had 478 hospitalisations and a 30-day readmission rate of 22.2 %, compared to 300 hospitalisations and a readmission rate of 7.0 % among matched controls (OR=3.79, 95 % CI 1.93-7.39). In the homeless cohort, having a primary care physician (OR=2.65, 95 % CI 1.05-6.73) and leaving against medical ad-vice (OR=1.96, 95 % CI 0.99-3.86) were associated with an increased risk of 30-day readmission. | Homeless patients had nearly four times the odds of being re-admitted within 30-daysas compared to low-income controls matched on age, sex and primary reason for admission to hospital. | Good |
| Miyawaki 2020 , United States | To determine whether homeless patients experience higher rates of readmissions and emergency department (ED) visits after hospital discharge than non-homeless patients, and whether the homeless patients exhibit lower rates of readmissions and ED visits after hospital discharge when they were admitted to hospitals experienced with the treatment of the homeless patients | longitudinal study , < 1 year | aged 18 or older, 134755 | | 51 | Deterministic linkage (using an unique identifier, such as a social security number) , Logistic regression model | | 30-day all-cause readmission, 30-day all-cause ED visit after hospital discharge. | Homeless patients had higher rates of readmissions and ED visits after hospital discharge. Homeless patients treated at homeless-serving hospitals exhibited lower rates of readmissions and ED visits after hospital discharge than homeless patients treated at non-homeless-serving hospitals. | Homeless patients were more likely to be readmitted or return to ED within 30 days after hospital discharge, especially when they were treated at hospitals that treat a small proportion of homeless patients. These findings suggest that homeless patients may receive better discharge planning and care coordination when treated at hospitals experienced with caring for homeless people. | Good |
| Lewer 2021 , UK | To compare the risk of hospital readmission among homeless inpatients with housed inpatients living in socioeconomically deprived areas. | Cohort study , 3 years | Homeless adults aged over 18, 2772 | 72 | 44.21 | Deterministic linkage (using an unique identifier, such as a social security number) , Negative Binomial model | | the counts of planned hospital readmissions, emergency hospital readmissions, and Accident and Emergency (A&E) visits | Homeless patients had 2.49 (95% CI 2.29 to 2.70) times the rate of emergency readmission, 0.60 (95% CI 0.53 to 0.68) times the rate of planned readmission and 2.57 (95% CI 2.41 to 2.73) times the rate of A&E visits compared with housed patients. The 12-month risk of emergency readmission was higher for homeless patient, and the risk of planned readmission was lower for homeless patients | Hospital patients experiencing homelessness have high rates of emergency readmission that are not explained by health. This highlights the need for discharge arrangements that address their health, housing and social care needs | Good |
| LaWall 2019 , United States | To examine whether variables for social isolation and homelessness as captured in a health systemâ ™s EHRs over2 years predicted 30-day potentially preventable readmission(PPR) | Cohort longitudinal study , 2 years | Homeless adults aged over 18, 2385 | 52.1 | NA | Deterministic linkage (using an unique identifier, such as a social security number) , Logistic regression model | | whether a person’s initial hospitalization during the study period resulted in a 30-day PPR | 4.2% (899) hospitalisations had a 30-day PPR. In bivariate analysis, living alone did not significantly affect likelihood of a 30-day PPR (16.6% [3,376 hospitalisations] without PPR vs 14.4% [128 hospitalisations] with PPR; P = .09). However, documented homelessness did show a significant effect on the likelihood of 30-dayPPR in the bivariate analysis (11.1% [2,259 hospitalisations] without PPR vs 14.1% [126 hospitalisations] with PPR; P = .006). Factors that were significantly associated with PPR were comorbid conditions, discharge disposition, and use of an assistive device | Homelessness predicted PPR in descriptive analyses. Neither living alone nor homelessness predicted PPR once other factors were controlled. Instead, indicators of physical frailty (i.e., use of an assistive device) and medical complexity (e.g., hospitalisations that required assistive care post-discharge, people with a high number of comorbid conditions) were significant. | Good |
| Khatana 2020 , United States | To evaluate the association of homelessness with readmission rates across multiple US states. | Cohort study , 6 years | Homeless individuals 18 years of age, 515,737 hospitalisations | 77.7 | 46.8 | Deterministic linkage (using an unique identifier, such as a social security number) , multivariate analyses | | Thirty- and 90-day readmission rates | After adjusting for cause of index hospitalization, state, demographics, and clinical comorbidities, 30-day and 90-day readmission rates were higher for index hospitalisations in the home-less compared with those in the housed group. The difference in 30-day readmission rates between homeless and housed groups was the largest in Florida (30.4% vs.19.3%; p< 0.001), followed by Massachusetts (23.5% vs.15.2%; p< 0.001) and New York (15.7% vs. 13.4%; p<0.001) (combined 17.3% vs. 14.0%; p< 0.001). Among the most common causes of hospitalization, 30-day readmission rates were 4.1 percentage points higher for the homeless group for mental illness, 4.9 percentage points higher for diseases of the circulatory system, and 2.4 percentage points higher for diseases of the digestive system | After adjusting for demographic and clinical characteristics, homelessness is associated with significantly higher 30- and 90-day readmission rates, with a significant variation across the three states. Effective and scalable interventions targeted towards the post-discharge period are urgently needed to address the disparities observed in our study. | Good |
| Doran 2013 , United States | To determine 30-day hospital readmission rates among patients who are homeless and examine factors associated with hospital readmissions in this population | Cohort study , < 1 year | homeless aged 18 or older, 113 | 72.6 | 48.9 | Fuzzy / Probabilistic linkage (linking by people's names etc) , Bivariate and multivariable analyses | | all-cause hospital readmission to the study hospital within 30 days of hospital discharge. | Half (50.8%) of all hospitalisations resulted in a 30-day hospital inpatient readmission and 70.3% resulted in either an inpatient readmission, observation status stay, or emergency department visit within 30 days of hospital discharge. Most read-missions occurred early after hospital discharge (53.9% within1 week, 74.8% within 2 weeks). Discharge to the streets or shelter versus other living situations was associated with increased risk for readmission in multivariable analyses. | Patients who were homeless had strikingly high 30-day hospital readmission rates. These findings suggest the urgent need for further research and interventions to improve post discharge care for patients who are homeless. | Good |
| **Care home admission and Shelter usage** | | | | | | | | | | | |
| Waldron 2019 , Ireland | To examine the patterns of emergency accommodation use by the homeless population in Dublin City. | Cross sectional study , 4 years | PASS system contacts, 12734 | 65 | 37 | Deterministic linkage (using an unique identifier, such as a social security number) , k-mean cluster analysis | | Emergency shelter use | A temporary cluster (78%) experienced a small number of homeless episodes for relatively short periods of time, while an episodic cluster (10%) experienced multiple homeless episodes also for a short period of time. The chronic cluster (12%) experienced a small number of homeless episodes but with long stays in emergency shelter. | The findings have implications for the operation of emergency homeless accommodation in Ireland and, in particular, the targeting of interventions and the re-directing of resources away from emergency accommodation responses towards a more effective emergency accommodation system for all stakeholders | Good |
| Chen 2021 , Canada | To compare homelessness pathways and housing outcomes between first-time and recurrent shelter users. | Cohort longitudinal study , 6 years | Homeless adults aged 25 or over, 255703 | 65.5 | 30 | Deterministic linkage (using an unique identifier, such as a social security number) , Logistic regression model | | housing status following a shelter exit | First-time users are over two times more likely to exit into newly acquired housing than recurrent users, and that the pathways into and out of homelessness vary significantly between the two groups. | The findings suggest that the composition of existing prevention strategies do not sufficiently meet the needs of first-time users experiencing financial and substance use challenges. For recurrent users, federal policies that promote Housing First initiatives increase exits into housing. However, duration of previous homelessness negatively influences housing outcomes, reinforcing the need for early intervention prevention initiatives. | Good |
| Byrne 2021 , United States | To examine the extent and timing of nursing home admissions among older adults who had their first visit at an emergency shelter or re-entered after an extended absence | Cohort longitudinal study , 6 years | Homeless adults aged 55+, 434 | 72.8 | 60.7 | Deterministic linkage (using an unique identifier, such as a social security number) , Cox-proportional hazards model | | Predictors of Nursing Home Admission | Roughly 12% of the study cohort had a nursing home admission within 4 years of their initial shelter entry and risk of shelter admission was highest in the first few months following shelter entry. Older age, diagnoses indicating alcohol use disorder, greater overall disease burden, and a prior history of nursing home admission were all associated with a higher risk of nursing home admission following shelter entry | Amidst ongoing growth in the number of older homeless adults, our study findings have important implications for efforts to meet the housing and health needs of this population | Good |
| **Other (employment rates, police crime victimisation)** | | | | | | | | | | | |
| Nilsson 2018 , Denmark | To study the risk of police-recorded crime victimisation in individuals with experiences of homelessness compared with the general population. | Cohort study , 15 years | people aged 15 years or older, 4286 | | NA | Deterministic linkage (using a unique identifier, such as a social security number) , Log-likelihood estimation. | | The first date of being a victim of any police-recorded crime. | Within the study period (Jan 1, 2001, to Dec 31, 2015), 1182749 individuals (9831776 person-years) aged 15 - 35 years were included, of which 184813 (15.6%) had at least one crime victimisation incident (73999 [40%] of which were violent victimisations). | Homeless populations are at substantially increased risk of crime victimisation, highlighting the need for strategic and targeted approaches to prevent homelessness and to help people out of homelessness. Improvements in multiagency working (such as between homeless shelters, health-care services, substance misuse services, and police forces) might be important to reduce the risk of victimisation in marginalised populations, such as those with complex psychiatric or social problems, with experience of homelessness. | Good |
| Cobb-Clark 2017 , Australia | To examine the long-run employment consequences of experiencing homelessness in childhood rather than later in life. | longitudinal study , 3 years | Individuals aged 21-54, 801 | | | Fuzzy / Probabilistic linkage (linking by people's names etc) , GLM | | adult employment | Those experiencing homelessness for the first time as children are less likely to be employed. For women, this relationship is largely explained by the lower educational attainment and higher welfare receipt (both in general and in the form of mental illness-related disability payments) of those experiencing childhood homelessness. Higher rates of high school incompletion and incarceration explain some of the link between childhood homelessness and employment; however, childhood homelessness continues to have a substantial direct effect on male employment rates. | Those experiencing homelessness for the first time as children are less likely to be employed. For women, this relationship is largely explained by the lower educational attainment and higher welfare | Fair |
