# Supplementary material for: Predictors and consequences of homelessness in whole-population observational studies that used administrative data: a systematic review
Source: BMC Public Health. 2023 Aug 24;23:1610. doi: 10.1186/s12889-023-16503-z (PMC10463451; doi:10.1186/s12889-023-16503-z)
Supplement: Supplementary file 2 — Additional file 2: Table S2. [file 12889_2023_16503_MOESM2_ESM.docx]

**Table S2**

**PICO criteria**

| **Criteria** | **Inclusion** | **Exclusion** |
| --- | --- | --- |
| **Population** | Individuals who meet at least one of the European Typology for Homelessness and Housing Exclusion (ETHOS) criteria: roofless, houseless, living in insecure housing, living in inadequate housing. | Individuals not classified as homeless |
| **Intervention** | Data administration/Record linkage studies reporting on the health and non-health outcomes of homeless | conference abstracts, reviews, comments and letters without original data |
| **Comparator** | Not applicable | Not applicable |
| **Outcomes** | **Health related outcomes:**  Mental health  Substance use  Use of healthcare services—including episodes of hospitalisation and use of emergency services.  Other, unanticipated measures of health and aspects of well-being associated with health and mental health.  **Non health related outcomes:**  Housing stability. | Self-reported health and quality of life—questionnaires and interviews recording perspectives |
| **Study design** | Data administration studies  Record linkage studies | Editorials, protocol papers, systematic reviews, literature reviews, methodology, descriptive studies (e.g., historical discussion and single case reports), non-English language studies. |
