# Supplementary material for: Predictors and consequences of homelessness in whole-population observational studies that used administrative data: a systematic review
Source: BMC Public Health. 2023 Aug 24;23:1610. doi: 10.1186/s12889-023-16503-z (PMC10463451; doi:10.1186/s12889-023-16503-z)
Supplement: Supplementary file 3 — Additional file 3: Table S3. [file 12889_2023_16503_MOESM3_ESM.docx]

# Table S3

# Detailed search strategy

Embase

1 homelessness/ or homeless person/ or homeless*.mp. 18034

2 Medical record linkage.mp. 378

3 administrative data.mp. 13658

4 record linkage.mp. 4906

5 discharge claim.mp. 8

6 discharge data.mp. 4849

7 hospital record.mp. 896

8 outpatient record.mp. 49

9 inpatient record.mp. 73

10 physician claim.mp. 54

11 Clinical Coding.mp. 743

12 admissions record.mp. 4

13 2 or 3 or 4 or 5 or 6 or 7 or 8 or 9 or 10 or 11 or 12 24700

14 1 and 13 197

15 transient living.mp. 8

16 street people.mp. 37

17 rough sleeping.mp. 27

18 rough sleeping.mp. 27

19 15 or 16 or 17 or 18 72

20 1 or 19 18053

21 13 and 20 197

Web of science

1. (((((TS=(homeless)) OR TS=(homelessness)) OR TS=(homeless*)) OR TS=(transient living)) OR TS=(street people)) OR TS=(rough sleeping)
2. (((((((((((TS=(Medical record linkage)) OR TS=(administrative data)) OR TS=(record linkage)) OR TS=(discharge claim)) OR TS=(discharge data)) OR TS=(hospital record)) OR TS=(outpatient record)) OR TS=(inpatient record)) OR TS=(physician claim)) OR TS=(Clinical Coding)) OR TS=(admissions record)) OR TS=(record linkage)
3. #1 AND #2

Medline

1 Homeless Persons/ or homeless.mp. 12647

2 transient living.mp. 6

3 street people.mp. 35

4 rough sleeping.mp. 21

5 1 or 2 or 3 or 4 12666

6 Medical record linkage.mp. or Medical Record Linkage/ 4853

7 administrative data.mp. 9982

8 record linkage.mp. 7514

9 discharge claim.mp. 4

10 data administrative.mp. 60

11 discharge data.mp. 3268

12 hospital record.mp. 567

13 outpatient record.mp. 24

14 inpatient record.mp. 36

15 physician claim.mp. 28

16 Clinical Coding.mp. or Clinical Coding/ 2471

17 admissions record.mp. 2

18 record linkage.mp. 7514

19 6 or 7 or 8 or 9 or 10 or 11 or 12 or 13 or 14 or 15 or 16 or 17 or 18 23253

20 5 and 19 142
